# Chemosensitization of Solid Tumors by Inhibition of Bcl-xL Expression Using DNAzyme

**DOI:** 10.18632/oncotarget.1996

**Published:** 2014-05-20

**Authors:** Xiaohui Yu, Lifang Yang, Murray J. Cairns, Crispin Dass, Edward Saravolac, Xiong Li, Lun-Quan Sun

**Affiliations:** ^1^ Center for Molecular Medicine, Xiangya Hospital, Central South University, Changsha, China; ^2^ Cancer Research Institute, Central South University, Changsha, China; ^3^ Schizophrenia Research Institute, Sydney, NSW, Australia and School of Biomedical Sciences, Faculty of Health and Medicine, University of Newcastle, NSW, Australia; ^4^ Health Science, School of Pharmacy, Curtin University, WA, Australia; ^5^ LCT Global, Auckland, New Zealand

**Keywords:** DNAzyme, bcl-xL, chemosensitization, apoptosis

## Abstract

DNAzymes are a novel class of gene suppressors that selectively bind to an RNA substrate by Watson-Crick base pairing and cleave phosphodiester bonds. To explore the potential for therapeutic use of catalytic DNA molecules, active DNAzymes targeting the bcl-xL gene were generated through a multiplex *in vitro* selection. The DNAzyme-mediated down-regulation of the bcl-xL expression was demonstrated in various cancer cell lines by Western blots. Treatment of the cells with the active DNAzyme led to increases in percentage of apoptotic cells and cytochrome c release from mitochondria, a hall marker of apoptosis. When combined with chemotherapeutics such as Taxol, the DNAzyme significantly sensitised a panel of cancer cells to apoptosis as measured by cell survival assay. In Taxol-resistant cells, down-regulation of bcl-xL expression by the DNAzyme reversed the chemo-resistant phenotype of the cancer cells. In a xenograft mouse model, the DNAzyme was delivered into the tumors via an ALZET osmotic pump and shown to chemosensitize PC3 tumor when treating with Taxol. The results from the present study demonstrate that bcl-xL DNAzyme treatment facilitates apoptosis in solid tumors and suggest the potential use of bcl-xL DNAzyme in combination with chemotherapeutics for cancer therapy.

## INTRODUCTION

Apoptosis is a complex process resulting in the regulated destruction of a cell, which plays a major role in normal development, cellular response to injury and carcinogenesis [1]. It has been suggested that apoptosis is one of the major mechanisms of cell death in response to cancer therapy [2]. The Bcl-2 family of proteins are among the most studied molecules in the apoptotic pathway. In this gene family, some are apoptosis inducers, including, bax, bak, bcl-Xs, bad, bid, bik and hrk, and others, such as bcl-2, bcl-XL, bcl-w, bfl-1, brag-1, Mcl-1 and A1 are apoptosis suppressors [3]. Bcl-2 or bcl-xL levels are elevated in a broad range of human cancers, indicating that these molecules may have a role in raising the apoptotic threshold in a broad spectrum of cancerous disorders. Expression of Bcl-xL in the NCI 60 cell lines strongly correlated with resistance to most chemotherapy agents [4]. It has been suggested that a decrease in Bcl-xL levels or the inhibition of Bcl-xL activity might provoke apoptosis or at least sensitise cells to apoptotic death. In the absence of a clearly defined biochemical mechanism of action or activity for this family of cell-death regulatory proteins (for which conventional inhibitors could therefore be developed), gene therapy and antisense approaches have become a reasonable alternative. For example, antisense oligonucleotides to bcl-xL have been shown to be active in down-regulation of the bcl-xL expression, leading to an increased chemosensitivity in a range of cancer cells [5].

An ideal oligonucleotide-based gene inactivation agent targeting RNA would combine the self-sufficient RNA digestion capability, such as “hammerhead” and “hairpin” ribozymes, with the relative biological resilience of the antisense oligodeoxynucleotides (ODN). Although DNA molecules with RNA cleavage activity have not been observed in nature, some have been derived as a result of an artificial evolutionary system known as *in vitro* selection [6-9]. In an *in vitro* selection system, DNA liberated from its complementary strand is free to explore a full range of structural possibilities, some of which have been found to be capable of catalytic activity, including site specific RNA cleavage and ligation [10, 11]. The 10-23 DNA enzyme or DNAzyme was named from its origin as the 23rd clone characterised from the 10th cycle of *in vitro* selection [10]. This enzyme has a number of features, which endow it with tremendous potential for applications both *in vitro* and *in vivo*. These include its ability to cleave almost any RNA sequence with high specificity provided it contains a purine-pyrimidine dinucleotide. This can be accomplished at very high kinetic efficiency with rates approaching and even exceeding those of other nucleic acid and protein endoribonucleases [10]. The ability of the 10-23 DNAzyme to specifically cleave RNA with high efficiency under simulated physiological conditions has fuelled expectation that this agent may have useful biological application in a gene inactivation strategy [12-16]. Recent studies showed promising clinical efficacy of the DNAzymes targeting c-jun and EBV-encoded oncogene for treating various cancers [17, 18], which demonstrated the feasibility to use DNAzymes as therapeutics.

Here, we identified an active DNAzyme against the bcl-xL gene by a multiplex selection. This DNAzyme was shown to effectively down-regulate the Bcl-xL expression in a range of cancer cells, thus overcome the anti-apoptotic block and sensitized the cancer cells to chemotherapy in xenograft animal models.

## RESULTS

### Identification of Cleavable sites in bcl-xL mRNA

**Figure 1:**
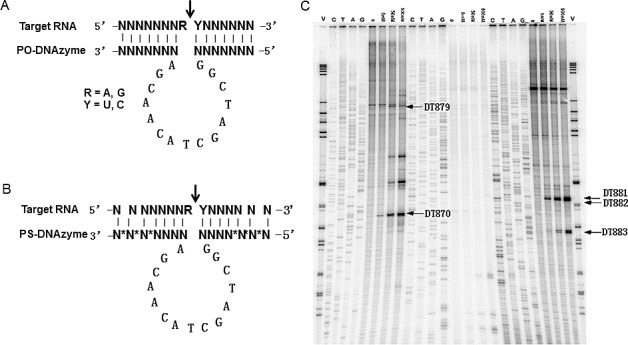
Schematic description of “10-23” DNAzyme A, unmodified DNAzyme (PO-DNAzyme); B, phosphorothioate-modified DNAzyme (PS-DNAzyme) as indicated by *. C, Multiplex *In vitro* selection of bcl-xL DNAzymes. Bcl-xl DNAzymes incubated with its RNA substrate for 60 minutes in the presence of 10 mM Mg^2+^ at 37^o^C. Primer extension was performed using the sequence-specific primers along the bcl-xl mRNA. The reactions were analysed alongside with DNA sequencing on a polyacrylamide gel. A representative gel is presented. V, molecular markers; C/A/T/G, sequencing ladders; 0/5/50/500 nM, a mixture of DNAzymes at the defined concentrations.

DNAzymes were designed based on “10-23” model (Fig 1A). A bcl-xL cDNA clone (926 bp) was produced from the RNA sample of PC3 cells and used for screening the potential cleavable sites of DNAzymes. By bioinformatics analysis, 81 potential AU or GU sites were found and subjected to analyses on the thermodynamic stability of the enzyme-substrate heteroduplex as predicted by the hybridisation free energy [19]. DNAzymes with the greatest heteroduplex stability indicated by a low free energy of hybridisation (calculated using the nearest neighbour method), was often found to have the greatest kinetic activity. As a result, 26 DNAzymes with Δ G°<- 16 kcal/mol were chosen for the further multiplex cleavage assay (sTable 1).

In order to select active DNAzymes, *in vitro* selection was performed using a multiplex method, which enables a pool of DNAzymes to be screened for their ability to access and cleave RNA substrate under simulated physiological conditions [20]. Based on the bioinformatics and physiochemical analyses as shown in sTable 1, twenty-six DNAzymes (0nM, 5nM, 50nM and 500nM) and RNA substrate (400nM) were incubated for cleavage reaction and primer extension was then performed with Superscript II reverse transcriptase to define the cleavage sites within the bcl-xL transcript. The sequencing ladders were used as a guide to attribute cleavage bands to specific DNAzymes. The relative cleavage strength of each DNAzyme was determined by intensity of the cleavage products. A representative gel was shown in Fig 1B. DNAzymes were ranked according to their cleavage ability at lowest concentration (5nM). The multiplex selection resulted in 10 active DNAzymes that could efficiently cleave the bcl-xL mRNA (sTable 1).

### Chemical modification of DNAzymes

**Figure 2:**
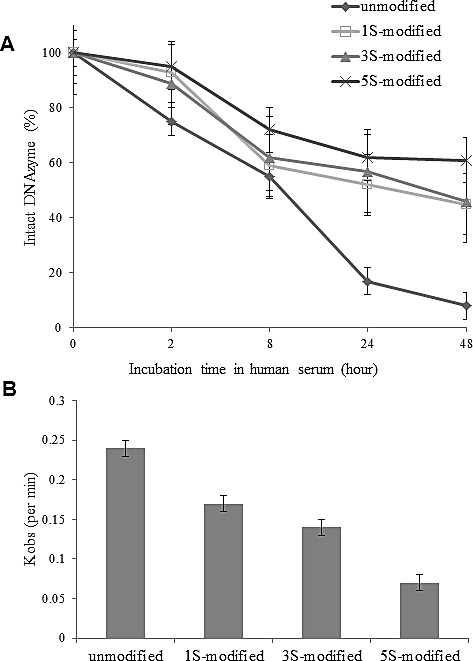
Effect of phosphorothioate-modification on DNAzyme stability in human serum and cleavage kinetics A: the modified DNAzymes were incubated with the serum, extracted at the defined time points, and ^32^P-labelled. Intact DNAzyme (%) was presented as a ratio of the band intensity at different time points to the band intensity at the 0 time point. B: the kinetic efficiency was measured under a single turnover condition and *k*_obs_ was used as an indicator for cleavage activity.

To increase DNAzyme stability in cells, 1-5 phosphorothioate (PS) linkages were incorporated into each of the arms in DNAzyme (PS-Dz, DT882). The DNAzymes' stability in human serum was significantly improved with the increase of number of PS linkages (Fig 2A). To examine if the modification had any impact on DNAzyme's catalytic activity, we further performed a single turnover kinetic analysis of the modified DNAzyme. As shown in Fig 2B, the PS modification had decreased the cleavage efficiency (*k*_obs_) of the modified DNAzymes, compared with the unmodified one. To strike a balance between the stability and catalytic activity, we selected the 3+3 PS modification scheme for the further cell and *in vivo* studies.

### Effect of bcl-xL DNAzymes on bcl-xL expression

**Figure 3:**
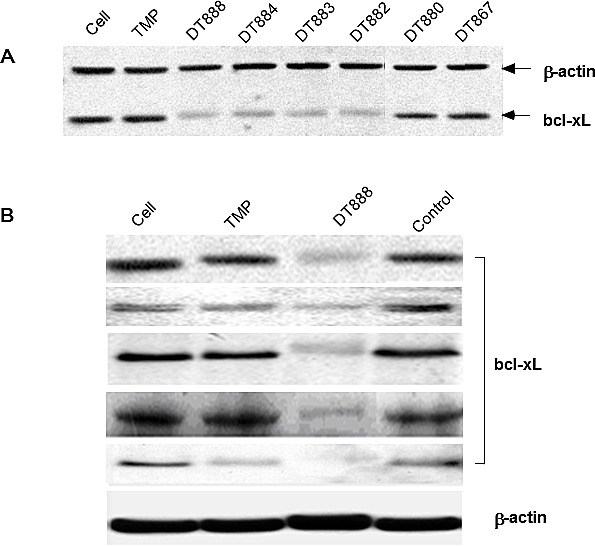
Inhibition of bcl-xL protein expression by bcl-xL DNAzymes A. Screening of the selected DNAzymes (2 μM) using Western blotting was performed in PC3 cells and a representative blot was presented. Bcl-xL antisense or a scrambled control were used as controls. B. Validation of inhibitory activity of Bcl-xL DNAzyme (DT882) in different cancer cells was performed in PC3 (prostate cancer), T24 (bladder cancer), A549 (lung cancer), CNE-1 (nasopharyngeal carcinoma) and HCT116 (colon cancer) cells. TMP, transfection reagent; Control, scrambled DT882.

Using the modified version of 16 DNAzymes derived from the multiplex selection, we performed further testing for their ability to down regulate the bcl-xL expression in cells. The assays were performed in PC3 cells (a prostate cancer cell line). After screening all 16 DNAzymes, three DNAzymes (DT 882, DT883, and DT884) exhibited a very strong inhibitory effect on bcl-xL protein expression as shown in a representative gel (Figure 3A). An antisense control (ctggatccaaggctctaggt) and a negative control (scrambled arms of DT882, tggtgtgtgggctagctacaacgagttaataaa) were used in the screening. Based on the results of *in vitro* cell screening, we next selected one of the most active DNAzyme DT882 for further validation of its activity of down-regulation of bcl-xL expression in a panel of cancer cell lines (PC3, prostate cancer; T24, bladder cancer; A549, lung carcinoma; CNE-1, nasopharyngeal carcinoma; HCT116, colon cancer). Figures 3B showed that anti-bcl-xL DNAzyme DT882 reduced the level of the bcl-xL gene expression in the cell lines tested, which indicated that DT882 could be effectively transfected into the cells, found its target mRNA and inhibited bcl-xL expression in cells.

### Induction of apoptosis by Bcl-xL specific DNAzyme

Having shown the DT882 inhibition of bcl-xL expression in cancer cells, its effect on apoptosis of PC3 cells was further examined. In the assay, same transfection procedure as in Western blotting assay was used, except those cells were subject to Annexin-V staining after the overnight incubation with the DNAzyme. Fig 4A clearly showed that there was a substantial increase in apoptosis population in the DNAzyme treated cells (23.17%), as compared with the control-treated (an inactivated DNAzyme with scrambled arm sequences of DT882), indicating that the cells treated with the bcl-xL DNAzyme was provoked to undergo apoptosis. To determine the effect of bcl-xL DNAzyme on the early events of apoptosis caused by down-regulation of bcl-xL, the measurement of Cytochrome C release from the cells was performed. As shown in Figure 4B, DNAzyme-mediated reduction of bcl-xL in PC3 cells led to an increased release of Cytochrome C. This result not only confirmed the data from FACS analysis, but also validated the specificity of the DNAzyme in induction of apoptosis via mitochondria pathway in PC3 cells.

### Chemosensitization of Cancer Cells with anti-bcl-xL DNAzyme

The Bcl-xL protein has been shown in a number of cell lines as a potent protector of cellular apoptosis induced by anti-neoplastic agents. To examine if DT882 could sensitise cancer cells to the effect of cytotoxic chemotherapy by down-regulating bcl-xL expression, cell survival was measured using MTS assays in a panel of cancer cells treated with either the DNAzyme alone or DNAzyme plus anti-cancer agent Taxol. The results showed that the anti-bcl-xL DNAzyme DT882 sensitised all the cancer cells to Taxol treatment (Table 1). This sensitization led to about 150~260% increase of cell death in a range of cancer cell lines treated with DT882 plus Taxol, compared with that treated with Taxol alone. To examine if DT882 could reverse the Taxol-resistance, a Taxol-resistant cell line CNE2R (nasopharyngeal carcinoma) were assayed for possible drug resistance reversal. As shown in Figure 4C, while the Taxol treatment in a range of concentrations did not cause cell death of the resistant cells or the control-treated resistant cells, DT882 treated resistant cells showed a significant reduction in cell survival, indicating that down-regulation of the bcl-xL in the Taxol-resistant cells could restore the cellular response to the chemotherapeutics.

**Table 1:** Chemosensitization of cancer cells to Taxol by bcl-xL DNAzyme DT882

Cancer Cells	Cell Death (%)*							Chemosensitization Index (%) #
	Scr Ctrl	Scr Ctrl+ Taxol	DT882	DT882+ Taxol	AS	AS+Taxol	Taxol	
PC3	1.75±0.27	16.33±3.10	23.17±4.11	46.51±6.66	19.33±2.07	38.51±3.33	15.20±2.10	205
T24	6.99±1.23	21.01±1.78	31.33±3.95	56.71±6.12	22.53±2.03	41.78±4.01	18.78±2.78	202
MDA-MB-231	3.10±0.45	12.09±2.11	19.88±3.28	39.89±3.51	18.64±2.83	35.02±3.44	10.90±1.11	266
CNE1	2.99±0.31	15.55±3.87	25.76±5.12	42.06±5.42	21.54±4.01	39.55±3.81	16.66±2.65	152
B9-58	1.44±0.64	13.67±2.45	19.66±3.33	41.15±6.65	19.01±2.11	37.47±2.60	11.76±3.10	250
HCT116	2.89±0.41	22.51±5.50	24.83±2.21	57.34±7.25	24.69±4.23	41.28±3.55	21.89±2.89	162
A549	4.67±0.87	19.23±2.99	19.88±1.56	42.78±2.11	21.22±3.34	39.56±6.01	17.44±2.67	145

* Cell death (%) was calculated as (untreated – treated)/untreated.

# Sensitization index was expressed as (DT882+Taxol – Taxol) / Taxol x 100.

**Figure 4:**
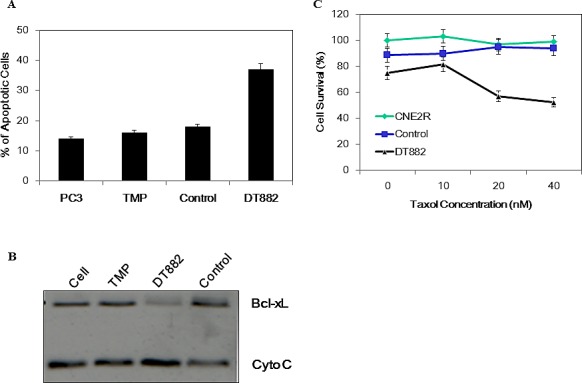
Induction of apoptosis by bcl-xL DNAzyme PC3 cells were transfected with DT882 and control. The cells were subjected to either Annexin-V assay by FACS (A) or Western blot assay for cytochrome C (B). TMP, transfection reagent; Control, scrambled DT882 control. C, a Taxol-resistant cell line of nasopharyngeal carcinoma (CNE2R) was used for MTS assays in the presence of a series of Taxol concentrations.

### Chemosensitization in human tumour xenograft model by anti-bcl-xL DNAzyme DT882

**Figure 5:**
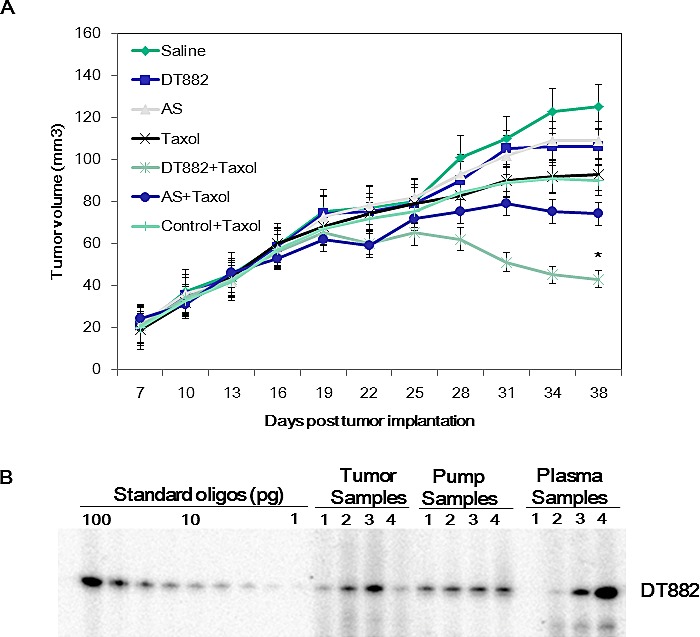
Chemosensitization of PC3 human xenograph prostate cancer by anti-bcl-xL DNAzyme A: Nude mice bearing established, subcutaneously growing PC3 tumour xenograft either remained untreated (saline) or were treated with DT882, antisense control, Taxol or DNAzyme + Taxol and AS+ Taxol. DNAzyme DT882 or AS was delivered using an osmotic pump and Taxol was administrated via i.p. route weekly. Tumour size was measured at the time points indicated. AS, antisense control (25). B: DNAzyme (DT882) was extracted from tumor tissues, plasma and the remaining solution of the pumps, labelled and assayed on PAGE.

In order to demonstrate if DNAzyme-mediated down-regulation of the bcl-xL expression resulted in chemosensitization of tumour cells to anticancer drugs *in vivo*, a murine model with human PC3 prostate cancer xenograft was used to determine the sensitivity to the chemotherapeutic. In the experiments, seven groups of mice (8 mice per group) (Saline, DNAzyme, AS, Control, Taxol, Taxol + DNAzyme, and Taxol + AS) were employed. When tumours reached an average volume of 100-200 mm^3^, an Alzet osmotic pump, which was used as a delivery vehicle for DNAzyme oligonucleotide in tumour bearing mice, was surgically implanted in the peritoneum of the mouse via the abdominal route. As shown in Fig 5A, the combinational use of DT882 and Taxol markedly inhibited PC3 tumour growth compared with the groups of DNAzyme alone, Taxol alone or control plus Taxol.

To examine the *in vivo* stability of the DNAzyme and the pump delivery efficiency, the DNAzyme oligonucleotide was extracted from the tumor tissues, plasma and the remaining solution of the pumps in the mice treated with DT882 plus Taxol (day 14), and labelled with γ--^32^P-ATP. Analysis showed that the DNAzyme was stable over the treatment period and the Alzet osmotic pump provided a consistent delivery of the DNAzyme *in vivo* (Fig 5B).

## DISCUSSION

DNAzymes have been shown to effectively suppress gene expression both *in vitro* and *in vivo* [21]. Limited therapeutic efficacy has been demonstrated in patients in studies targeting c-jun and EBV-LMP1 [17, 18]. In the present report, we present a comprehensive study in which an active DNAzyme targeting bcl-xL gene was identified and validated through *in vitro* selection to *in vivo* testing, showing clear experimental evidence that the Bcl-xL DNAzyme could be an effective chemo-adjuvant agent for potential clinical applications in cancer chemotherapy.

Targeting a DNAzyme to a particular mRNA is generally a multi-step process. In our previous study, a multiplex selection approach was devised to facilitate the selection of the accessible sites for DNAzymes within the target mRNA [20], but only at the *in vitro* level. Here, we successfully applied this methodology and further proved that there was a consistency between the *in vitro* cleavage activity and *in vivo* efficacy of the DNAzymes. Thus, our study provided a streamline approach to therapeutic uses of DNAzymes.

Despite the range of anti-cancer studies using nucleic acid-based agents, one common factor necessary for the success of therapeutic uses of those agents is the ability to delivery of the agents in a precise and dependable manner. The antitumor effects of the therapeutic agents are influenced by schedule of drug administration. In majority of the studies testing anti-cancer oligonucleotides, intra-tumoral injection has been the method of the choice to deliver oligonucleotides [22], which was somewhat invasive and less efficient. In this study, we surgically implanted the ALZET osmotic pump adjacent to the tumor, permitting continuous, controlled dosing, thus enabling us to achieve steady state conditions and accurate delivery of the DNAzyme over prolonged periods of time and to show a significant effect of chemosensitization by the DNAzyme to Taxol treatment.

To date, there have been substantial amount of preclinical and clinical studies in search for the inhibitors of Bcl-2 family, among which one Bcl-2 antisense and three small molecule inhibitors are being tested in clinical trials [23, 24]. However, it has been well recognized that the agents with high specificity tend to induce cancer drug resistance, while broader acting agents could cause unwanted toxicity. Considering that the anti-apoptotic Bcl-2 family is a large and redundant family of proteins, some of which have been shown to contribute to drug resistance in cancer cells [25, 26], inhibition of multiple Bcl-2 family members would be necessary in order to optimize the therapeutic effect. Importantly, previous studies showed that paclitaxel (Taxol) causes phosphorylation and inactivation of Bcl-2 [31-34]. Phosphorylation of Bcl-2 takes place only during mitotic arrest and the duration of the arrest is important for cell killing by paclitaxel/Taxol [35, 36]. However, Bcl-2 can be substituted by Bcl-xL [37], which underscores the importance to decrease Bcl-xL levels in Taxol-treated cells. This decrease actually increases sensitivity to Taxol as now shown by current work. Thus, our strategy further confirm that one anti-apoptotic protein can be substituted by the other, providing evidence that the Taxol resistance can be overcome by targeting Bcl-xL.

In conclusion, we present a novel agent that catalytically cleave the Bcl-xL mRNA, one of the important members of Bcl-2 family, thus add another candidate of inhibitors of anti-apoptotic Bcl-2 family with new mechanisms for potential multimodality treatment of cancers.

## MATERIALS AND METHODS

### Cell cultures

PC3 (prostate adenocarcinoma), T24 (bladder carcinoma), MDA-MB-231 (Breast cancer), A549 (lung carcinoma), B9-58 (lymphoma) and HCT116 (colon carcinoma) human cancer cell lines were obtained from the American Type Culture Collection. CNE1 (nasopharyngeal carcinoma) is the EB virus negative low differentiated nasopharyngeal squamous carcinoma cell line [27]. All cell lines were maintained according to vendors' recommendations.

### DNAzyme synthesis

All the oligonucleotides were made by TriLink BioTechnologies (San Diego, CA) and purified by gel electrophoresis for *in vitro* studies and by HPLC for cell-based assays and *in vivo* studies.

### Design and thermodynamic analysis of DNAzymes

DNAzyme sequences for each target are assembled using the 10-23 catalytic motif [ggctagctacaacga] and hybridising arms specific for each site along the target RNA transcript (Fig. 1A). The length of each arm is usually fixed at 9 bases; however, these can be shortened or lengthened depending on their individual predicted hybridisation free energy. Each DNAzyme oligonucleotide is designed to target purine-uracil (RU). In most cases we ignore purine-cytosine sites as in our experience they are less reactive than RU sites, particularly AC junctions [28]. The potential DNAzymes were subjected to thermodynamic analyses (hybridization free energy and Tm) [19].

### Multiplex selection of active DNAzymes *in vitro*

DNAzyme target site selection was described previously [20]. Briefly, linear template DNA for the production of the bcl-xL transcript was prepared by PCR in a mixture containing 10 pM cDNA, 1 μM of the forward T7 phage promoter primer [TAATACGACTCACTATAGGGAGA-AAGATTCTGAAGGGAGAG] and 1 μM of the reverse primer [CGGGTTTCTCCTGGTGGCAATG]. The reaction was carried out over 25 temperature cycles at 95oC for 30 s, 60oC for 90 s, and 72oC for 60 s in a solution containing 16.6 mM (NH4)2SO4, 67 mM Tris-HCl (pH 8.8), 6.7 mM MgCl2, 0.87 units of Ampli*Taq* DNA polymerase (Perkin Elmer) and 300 μM each of dGTP, dATP, dTTP, dCTP. PCR products were electrophoresed in a 1% agarose gel alongside appropriate size/ mass standards and visualised by ethidium fluorescence. The illuminated amplicon bands were then excised from the agarose slab and extracted using Gene Clean (Bio 101). Template DNA was then redissolved in diethyl pyrocarbonate (DEPC) treated RNase free water and the concentration adjusted to approximately 100ng.μl^−1^.

For RNA transcription, reaction was performed with 100 ng of purified template DNA and T7 RNA polymerase at 37oC for 3 h using an RNA transcription kit (Epicentre). After the 3 hour incubation the DNA template was degraded by the addition of 1 MBU of RNase-free DNase I and further 15 min incubation at 37^o^C. This reaction was then terminated by adding an equal volume of phenol/ chloroform/ isoamyl alcohol. The RNA contained in the aqueous phase could be used directly in the multiplex cleavage reaction or precipitated in 0.3M sodium acetate and two volumes of absolute ethanol.

Multiple DNAzyme oligonucleotides (0.5 nM-0.5 μM) and synthetic RNA substrate (0.2 μM) were pre-equilibrated separately for 10 min at 37oC in equal volumes of 50 mM Tris-HCl, pH 7.5, 10 mM MgCl2, 150 mM NaCl and 0.01 % SDS. The reaction was then initiated by mixing the DNAzymes and substrate together. After an hour the reactions were stopped by emersion in ice and precipitation in 0.3 M sodium acetate and two volumes of ethanol.

Primer extension analysis was then performed on DNAzyme cleaved RNA using SuperScriptII reverse transcriptase (Life Technologies). Two primers were designed for the extension reactions along the bcl-xL RNA [PE1: GTTTCTCCTGGTGGCAATG; PE2: CCATCCCGGAAGAGTTCA]. In each reaction 2 pmol of ^32^P-labelled primer was combined with 400 nM of RNA and denatured at 90oC for 5 min. For extension with Superscript reverse transcriptase, the primer was allowed to anneal slowly between 65oC and 45oC before adding the 1st strand buffer, dithiothreitol, deoxyribonucleotides and enzyme (according to the manufacturer's instructions). This mixture (final volume 20 μl) was then incubated for 1h at 45oC before being transferred to ice.

Sequencing fragments corresponding to each segment of the target were also generated by primer extension on the double stranded DNA template in the presence of chain terminating dideoxynucleotides (ddNTP). In these four reactions the dNTP concentration was reduced to 2.5 μM while being supplemented by either 10 μM ddGTP or 100 μM ddATP or 200 μM ddTTP or 100 μM ddCTP in 16.6 mM(NH4)2SO4, 67 mM Tris-HCl (pH 8.8), 6.7 mM MgCl2, 0.87 units of *Taq* DNA polymerase and 1.0 pmol of the 32P-labelled primer. This reaction was performed as a linear amplification over 25 temperature cycles at 95o C for 30 s, 60° C for 90 s, and 72° C for 60 s.

After primer extension, samples were combined with an equal volume of stop buffer (formamide/EDTA/loading dye) before electrophoresis on a 6% denaturing polyacrylamide gel. The corresponding image could then be revealed and the band intensity quantified at each position with Phosphorimager and ImageQuant software (Molecular Dynamics).

### DNAzyme stability assay and kinetic analysis

The DNAzyme (DT882) was modified with 1, 3 or 5 phosphorothioate (PS) linkages respectively. Unmodified and PS-modified DNAzymes were incubated at a final concentration of 10 μM in 100% human AB serum (Sigma, St. Louis, MO) at 37°C. Aliquots (10 μl) of the incubation mixture were removed at 0, 2, 8, 24 and 48 hours and diluted in 290 μl of 10 mM Tris/1 mM EDTA, pH 8.0, buffer (TE buffer). The diluted DNAzyme was extracted by sequentially adding 150 μl phenol and 150 μl chloroform with vortexing. Samples were centrifuged at 11,300*g* for 10 minutes, and 100 μl supernatant was collected. Aliquots of the aqueous supernatant were labeled with ^32^P and electrophoresed on a 16% polyacrylamide gel. Labeled DNAzyme bands were quantified using Molecular Dynamics ImageQuant software (Sunnyvale, CA), and the recovered DNAzyme was expressed as a percentage of the DNAzyme diluted directly into TE buffer.

The *in vitro* activity of DNAzymes was determined by measuring the rate of RNA cleavage under single turnover condition. The ^32^P-labelled RNA transcripts to be used in the cleavage reaction were produced utilising the RNA transcription kit (Epicentre). The transcripts were electrophoresed on a 16% denaturing PAGE and bands were excised and gel purified. The observed constant (k_obs_) is the rate of cleavage and it was calculated by regression analysis of the progress curve using the following equation:

[P] = [P]∞(1 - e^−1kobst^)

where [P] is the cleavage yield, [P∞] is the final cleavage yield, and t is the reaction time [29]. Cleavage reactions were performed over multiple time points.

### DNAzyme transfections

To facilitate delivery of DNAzyme oligonucleotides into cells, a cationic porphyrin, tetra meso (4-methylpyridyl) porphyrin (TMP), was used as a transfection reagent for intracellular delivery [30]. Briefly, 0.8 × 10^5^ cells were seeded in a 60-mm culture dish and incubated at 37^o^C, 5% CO_2_ overnight. DNAzyme and control molecules were transfected into cells with TMP at a charge ratio of 3. The final concentration of DNAzyme in the transfection mix was 2 μM in 2 ml of medium without serum. After 4 hours, the cells were replaced with fresh culture medium. Twenty-four hours after transfection, the cells were harvested for Western blot and cell biology analyses.

### Western analysis

Protein was extracted from the transfected cells in RIPA buffer (50 mM Tris-HCl, pH 8, 150 mM NaCl, 0.1% SDS, 1% NP-40, 0.5% sodium deoxycholate, 0.57 mM PMSF and 1 μg/ml aprotinin). Protein samples (40 μg) were run on 12% SDS-PAGE gels (BioRad), transferred to nylon membranes and immunoblotted with the following antibodies: anti Bcl-xL mouse monoclonal antibody (H-5, Santa Cruz), anti-Cytochrome C rabbit polyclonal antibody (H-104, Santa Cruz) and anti-β-actin monoclonal antibody (AC-74, Sigma). Binding of primary antibodies was detected with goat anti-rabbit or anti-mouse horse radish peroxidase conjugated secondary antibodies (Santa Cruz). This binding was visualized with the ECL western blotting detection reagents (Amersham Pharmacia Biotech, England) on exposure to chemiluminescent film.

### Apoptosis and cell survival assays

To determine apoptosis induced by DNAzymes, Annexin-V-FUOS Kit was used to detect apoptotic cells by FACS, according to the manufacturer's instruction (Roche). Apoptotic cells were quantified using a FACScan flow cytometer (Becton–Dickinson).

Chemosensitization effect of DNAzymes on cancer cells was examined using MTS assay. Cancer cells were treated with DNAzyme/TMP complex for 4 hours. The medium was then replaced with fresh DMEM containing 10% FBS and 100 nM Taxol, and further incubated for 72 hours. MTS assays were performed for cell viability of all the samples.

### Detection of cytosolic Cytochrome C expression in PC3 cells

Transfected cells were harvested by centrifugation at 200 *g* for 5 minutes at 4° C. The cell pellets were washed once with ice-cold phosphate-buffered saline (PBS) and resuspended with 5 volume of buffer containing 20 mM HEPES-KOH, pH 7.5; 10 mM KCl; 1.5 mM MgCl2; 1 mM sodium EDTA; 0.1% SDS; 1 mM dithiothreitol; and 0.1 mM phenylmethyl sulfonyl fluoride containing 250 mM sucrose. The cells were homogenized with a 22-gauge needle, and the homogenates were centrifuged at 1000*g* for 10 minutes at 4°C to remove nuclei, unbroken cells, and large membrane fragments. The supernatant was centrifuged once more at 10,000*g* at 4 °C for 30 min. The supernatant cytosolic fraction was collected, quantitated and used in Western analysis.

### *In vivo* mouse model and DNAzyme treatment

Four-week-old female Balb/C nude mice were obtained from the Animal Resources Center (Perth, Australia) and were housed and maintained in compliance with the National Health and Medical Research Council Statement on Animal Experimentation and the requirements of the New South Wales State legislation. Four groups of mice (8 mice per group) (Saline, DNAzyme, Taxol, Taxol + DNAzyme) were employed. At day 1: acclimatised nude male Balb/C athymic mice were injected with 1x 10^6^ PC3 cells suspended in 0.1 ml Matrigel in the right hind leg under methoxyfluorane anesthesia. Tumour growth was measured twice weekly using digital callipers and tumour volume is calculated using the (l * w * h* π/6) formula. When tumours reached an average volume of 100-200 mm^3^, an Alzet osmotic pump (BioScientific), which was used as a delivery vehicle for DNAzyme oligonucleotide in tumour bearing mice, was surgically implanted in the peritoneum of the mouse via the abdominal route. The Alzet model1002 pump is a capsule shaped pump (1.5 × 0.6 cm) and delivered a total volume of 0.5 ml at a rate of 0.25 μl/hr over a period of 14 days. The pump was filled with a saline solution containing DNAzyme oligonucleotide, which resulted in a dose rate of 12.5 mg/kg/d. Some mice received sub-optimal doses of 25 mg/kg Taxol by intraperitoneal route in a 200μl injection once weekly post-surgery for the duration of the study. At the end of 14d, mice were euthanized by cervical dislocation and tumours harvested. Tumours were weighed, snap-frozen and stored at –80^o^C till analysis. To analyse the concentration of the DNAzyme, the DNAzyme oligonucleotide was extracted from tumor tissue, plasma and remaining solution in the pumps with phenol/chloroform and precipitated with ethanol. The extracted DNAzyme was labelled with γ-^32^P-ATP by T4 polynucleotide kinase and assayed on PAGE.

### SUPPLEMENTARY TABLE


