# Supplementary material for: Chemosensitization of Solid Tumors by Inhibition of Bcl-xL Expression Using DNAzyme
Source: Oncotarget. 2014 May 20;5(19):9039–48. doi: 10.18632/oncotarget.1996 (PMC4253417; doi:10.18632/oncotarget.1996)
Supplement: Supplementary file 1 [file oncotarget-05-9039-s001.pdf]

Chemosensitization of Solid Tumors by Inhibition of Bcl-xL Expression Using DNAzyme

Supplementary Material

Sup Table 1: Design, thermodynamics and *in vitro* activity of bcl-xL DNAzymes.

DNAzyme Codes	DNAzyme Sequences	-G^o (kcal/mol)	*In vitro* Activity*
DT861	aagagttcaggctagctacaacgatcactacct	21.70	+
DT862	tttaccccaggctagctacaacgacccggaaga	27.70	+
DT863	cccagtttaggctagctacaacgacccatcccg	30.10	+
DT864	acaatgcgaggctagctacaacgacccagttta	23.60	-
DT865	aggccacaaggctagctacaacgagcgacccca	30.40	-
DT866	aaaaggccaggctagctacaacgaaatgcgacc	22.70	-
DT867	ttccacgcaggctagctacaacgaagtgccccg	30.90	-
DT868	cgctttccaggctagctacaacgagcacagtgc	27.60	+
DT869	ccttgtctaggctagctacaacgagctttccac	25.80	++
DT870	atacctgcaggctagctacaacgactccttgtc	25.60	+++
DT871	tcaccaataggctagctacaacgactgcatctc	22.50	++
DT872	actcaccaaggctagctacaacgaacctgcatc	24.80	-
DT873	tccgactcaggctagctacaacgacaatacctg	23.20	-
DT874	gcgatccgaggctagctacaacgatcaccaata	24.40	+
DT875	aagctgcgaggctagctacaacgaccgactcac	25.40	+
DT876	aagtggccaggctagctacaacgaccaagctgc	27.10	++
DT877	aggtggtcaggctagctacaacgatcaggtaag	22.80	++
DT879	tctcctggaggctagctacaacgaccaaggctc	27.10	+++
DT880	acaaaagtaggctagctacaacgacccagccgc	25.20	-
DT881	gtctggtcaggctagctacaacgattccgactg	25.40	++
DT882	tttttataaggctagctacaacgaagggatggg	18.90	++
DT883	acatttttaggctagctacaacgaaatagggat	17.40	+
DT884	tctgagacaggctagctacaacgattttataat	16.80	+
DT885	gctctgagaggctagctacaacgaatttttata	19.40	+++
DT886	agtcaaccaggctagctacaacgacagctcccg	27.50	++
DT887	gtggctccaggctagctacaacgatcaccgcgg	30.50	-

**In vitro* cleavage activity was assessed according to the band intensity at the lowest concentration (5nM). The DNAzymes with the activity> ++ were selected as efficient cleavers.
